# Factors associated with the practice of and intention to perform female genital mutilation on a female child among married women in Abakaliki Nigeria

**DOI:** 10.1186/s12905-023-02537-3

**Published:** 2023-07-17

**Authors:** Cosmas Kenan Onah, Edmund Ndudi Ossai, Okechukwu Matthew Nwachukwu, Gloria Ekwutosi Nwankwo, Hyacinth Ogbonna Mbam, Benedict Ndubueze Azuogu

**Affiliations:** 1 Department of Community Medicine, Alex Ekwueme Federal University Teaching Hospital, Abakaliki, Ebonyi State Nigeria; 2 Department of Community Medicine, Ebonyi State University, Abakaliki, Nigeria

**Keywords:** Female genital mutilation, Female genital cutting, Female circumcision, Harmful practice, Violation of human rights, Female sexual rights, Abakaliki Nigeria

## Abstract

**Background:**

Female Genital Mutilation (FGM), also known as Female Genital Cutting or Female Circumcision is the harmful excision of the female genital organs for non-medical reasons. According to WHO, approximately 200 million girls and women have been genitally mutilated globally. Its recognition internationally as human rights violation has led to initiatives to stop FGM. This study investigated factors associated with the practice and intention to perform FGM among married women.

**Methods:**

A cross-sectional study was conducted among 421 married women from communities in Abakaliki Nigeria. The participants were selected through multistage sampling. Data were collected through the interviewer’s administration of a validated questionnaire. Data were analyzed using IBM-SPSS version 25. Chi-square and logistic regression tests were employed to determine factors associated with the practice and intention to perform FGM at a p-value of ≤ 0.05 and confidence level of 95%.

**Results:**

The mean age of respondents is 40.5 ± 14.9 years. A majority, 96.7% were aware of FGM. On a scale of 15, their mean knowledge score was 8.1 ± 4.3 marks. Whereas 50.4% of the respondents were genitally mutilated, 20.2% have also genitally mutilated their daughters, and 7.4% have plan to genitally mutilate their future daughters. On a scale of 6, their mean practice score was 4.8 ± 1.2 marks. The top reasons for FGM are tradition (82.9%), a rite of passage into womanhood (64.4%), suppression of sexuality (64.4%), and promiscuity (62.5%). Women with at least secondary education are less likely to genitally mutilate their daughters (Adjusted Odds Ratio [AOR] = 0.248, 95% Confidence Interval [CI] = 0.094–0.652). Women who are genitally mutilated are more likely to genitally mutilate their daughters (AOR = 28.732, 95% CI = 6.171–133.768), and those who have previously genitally mutilated their daughters have greater intention to genitally mutilate future ones (AOR = 141.786; 95% CI = 9.584–209.592).

**Conclusions:**

Women who underwent FGM have a greater propensity to perpetuate the practice but attaining at least secondary education promotes its abandonment. Targeted intervention to dispel any harboured erroneous beliefs of the sexual, health, or socio-cultural benefits of FGM and improved public legislation with enforcement against FGM are recommended.

## Background

Female Genital Mutilation (FGM), also known as Female Genital Cutting or Female Circumcision is the partial or total removal of external female genitalia or other injuries to the female genital organs for non-medical reasons [1]. The World Health Organization (WHO) classifies FGM into four types: type I (clitoridectomy), type II (excision), type III (infibulation) and type IV which comprises all other harmful procedures performed on the female genitalia like pricking, piercing, incising, scraping, and cauterization [2]. The WHO estimates that more than 200 million girls and women alive have undergone FGM and more than 3 million girls are at risk of it annually, in about 30 countries in Africa, the Middle East, and Asia where FGM is rampant [1, 3, 4]. Despite that FGM prevalence has fallen in many countries among younger girls aged 15–19 compared to older women aged 45–49 [5, 6], the incidence remains high in many countries where it is practiced. For instance, a previous report of national FGM prevalence among women aged 15–49 years in West African countries showed high rates of 96.9%, 89.6%, and 82.7% respectively in Guinea, Sierra Leone, and Mali [5].

According to the Nigeria Demographic Health Survey 2018 (NDHS 2018), the national prevalence of FGM among women aged 15–49 years is 20% and the practice is on the rise among girls aged 0–14, the rates having risen from 16.9% in 2013 to 19.2% in 2018. [7] The regional prevalence in the three major tribes of Yoruba, Igbo and Hausa stands at 34.7%, 30.7% and 19.7% respectively and in terms of the geopolitical zones, the prevalence is highest in the South East (35%) followed by the South West (30%), and lowest in the North East (6%) zones. In Ebonyi State in the South East zone, the prevalence is 53.2%, seconding Imo State with the highest prevalence of 61.7% in the zone [7]. Previously, Lawani et al. reported that majority (66.3%) of the primigravid women in their survey in Abakaliki, the Ebonyi State Capital, had undergone FGM [8].

The practice of FGM has been defended upon socio-cultural, traditional, political, religious, and economic backgrounds [9–11]. Some of the socio-cultural reasons include cultural identity, female cleanliness, protection of virginity, improvement of fertility, prevention of immorality, better marriage prospects, and greater pleasure for the husband [1, 5, 9, 10]. FGM is often believed to reduce a woman’s libido and this is considered to help her resist illicit sexual intercourse and preserve marital fidelity [1]. FGM is also often seen as a necessary ritual for initiation into womanhood and is linked to cultural ideals of femininity and modesty [9, 10]. Further, the practice is seen in many cultures as a requirement for marriage, social inclusion, and approval [5, 9]. Consequently, family pressure to conform to the practice is a strong motivation to continue with FGM, and women who depart from the societal norm may face condemnation, harassment, and rejection [1, 12].

FGM has no health benefits, and it harms girls and women in so many ways [1] due to immediate and long-term physical, mental and psychosocial health consequences [2, 3, 6, 10]. Girls who undergo FGM face short-term complications such as severe pain, shock, excessive bleeding, infections, and difficulty in passing urine. In a long term, the victims are significantly at risk of adverse gynaecologic and obstetric outcomes like dyspareunia, prolonged labour, perineal tears, caesarean section, postpartum haemorrhage, episiotomy, extended maternal hospital stay, resuscitation of the infant, inpatient perinatal death and dysuria and the risks are greater with more extensive FGM [8, 13–16]. The treatment of FGM in 27 high-prevalence countries has been estimated to cost 1.4 billion United States Dollars (USD) per year and is projected to rise to 2.3 billion USD by 2047 if no action is taken [1].

Female genital mutilation is internationally recognized as a violation of the human rights of girls and women [1, 2, 5, 6]. The United Nations strives for the full eradication of the practice by 2030, with its inclusion as target 5.3 in the Sustainable Development Goal (SDG) [17]. In Nigeria, FGM is criminalized; offenders are punishable with a prison sentence of up to 4 years and or a fine of up to 200,000 Naira (US$480) or both [5, 18]. Similarly, FGM is prohibited in Ebonyi State with a State Law [19] that prescribes the same penalties for offenders as obtained in the federal law. Despite the enactment of these laws and the growing disapproval globally, the incidence of FGM remains high, raising questions about the factors behind the perpetuation of the practice. This study, therefore, investigated factors associated with the practice of and the intention to perform FGM on a female child among married women, intending to inform strategic interventions to curtail the harmful practice.

## Methods

### Study area and design

The study was conducted in Abakaliki Local Government Area (LGA) using a cross-sectional analytic design. Abakaliki is one of the 13 LGAs in Ebonyi State in the South East of Nigeria. The LGA, with a projected population of 198,793 [20], is made up of seven communities including six which are majorly rural and one which is majorly urban, accommodating part of Abakaliki, the capital of Ebonyi State. The people of the LGA engage in diverse occupations with a significant proportion of farmers. The population comprises predominantly Igbo ethnic group who practice Christianity. According to the 2015 Nigeria Education Data Survey, Ebonyi State had a female literacy rate of 40.3%, with only 11.1% of the females who completed secondary education and 4.3% who had more than secondary education. [21].

In terms of healthcare, there are 61 formal health facilities in Abakaliki LGA comprising 29 public and 32 private facilities, [22] and a majority of these facilities offer a wide range of maternal health services. The public health facilities include 2 tertiary health facilities (Alex Ekwueme Federal University Teaching Hospital Abakaliki and National Obstetric Fistula Centre Abakaliki) which offer specialist obstetric and gynaecologic services, one general hospital, 20 health centres and 6 health posts. However, the service delivery is very poor especially in the primary and secondary levels because of poor facilities, inadequate manpower and poor logistics. [22].

### Study population, sample size, and sampling technique

The study involved married women. A sample size (n) of 422, including anticipated non-response rate of 10%, was estimated using the Cochran formula (n = Z_α_^2^pq/d^2^) for sample proportion [23], with a standard normal deviate (Z_α_) of 1.96, a prevalence of FGM (p) of 49.6% reported in an earlier study [24], and a precision (d) of 5%. The respondents were selected using multistage sampling method. Firstly, four out of the seven communities including Nkaliki, Mgbabor, Agbaja-Unuhu, and Inyimagu-Unuhu were selected by balloting. Using estimated populations for each of the community, the sample size was allocated proportionately to the four communities, coming up to 95, 105, 109, and 113 respectively.

Households were selected from the communities using systematic sampling technique. Firstly, a list of the households in each of the communities was obtained from the Disease Surveillance and Notification Unit of the Health Department of Abakaliki LGA. A sampling interval (nth interval) was determined by dividing the number of households in each community by the sample size estimated for that community. Strategic locations in the communities were identified with the help of resource persons who were members of the communities. These places included a village square, two schools, and a market. In each of the communities, a pen was spined at the location and the street closest to and in the direction of the tip of the pen was followed to locate the first house on either the right or left side of the street, where the first household and first respondent was selected for interview. The spinning of the pen was done to make a random start. After the random start, subsequent households were selected using the nth interval. Where a street divided into two or more, the one on the right-hand side, which was predetermined, was followed.

All married women met in the houses visited were screened for eligibility to participate. The inclusion criterium was being legally married, whether living together with husband or not while the exclusion criteria were being divorced or widowed. However, only one woman was selected per household. In a polygamous family with more than one woman who met the inclusion criterium, one of the women was selected by balloting. This was done to ensure better representation of the dispositions of spouses of the married women to FGM. Using the same technique in all the communities, participants were selected until the sample size for each community was achieved.

### Data collection and analysis

Data was collected with a pre-tested, structured questionnaire, which was administered by the researchers. The questionnaire had four sections: (1) sociodemographic characteristic, (2) knowledge of FGM with 15 questions, (3) practices about FGM with six questions, and (4) reasons for performing FGM with ten questions. A mix of English and Igbo languages were used during the data collection. The choice of language used was left to the discretion of the participants; for those who were literate, English language was majorly used. For those who were not literate and could neither understand nor speak English language, they were interviewed in Igbo language. All the research team members who participated in the data collection are fluent in both English and Igbo languages, and they carried out pre-data collection rehearsals of the questions after the pretesting of the tool. Data collection lasted for eight days, at two days per community.

Data were entered into the IBM-SPSS version 25, cleaned, and transformed into variables of interest by recoding. Descriptive analyses were carried out using frequency, mean and standard deviation. Inferential statistics were done using chi-square at p-value of ≤ 0.05, and logistic regression of independent variables that had significant relationship with the outcome variables after bivariate analyses, were carried out at confidence level of 95%.

### Measurement of variables

Knowledge of FGM was assessed using fifteen variables (Table 1). A correct answer for each of the variables attracted a score of 1 while an incorrect answer was scored 0, giving a maximum knowledge score of 15 marks. Good knowledge of FGM was determined as the proportion of respondents who scored up to the mean mark (8.1 ± 4.3) while those that scored below the mean were categorised as having poor knowledge. The categorization of knowledge into good and poor was arbitrarily done to enable a test of the relationship of knowledge with the practice and the intention to perform FGM among the respondents.

**Table 1:** Knowledge of FGM, its side effects and complications

Variables	Frequency n = 421	%
**Aware of FGM**	407	96.7
**Knowledge about FGM**		
FGM is a violation of the fundamental human rights of girls and women.	252	59.9
FGM has no health benefit.	238	56.5
Genitally mutilated women are more likely to catch tetanus and sexually transmitted diseases like HIV.	224	53.2
FGM can reduce fertility in women.	157	37.3
FGM can cause prolonged labor during childbirth.	159	37.8
Side effects and complications of FGM*		
Severe bleeding	300	71.3
Reduction in sexual feeling	279	66.3
Pain during sexual intercourse	208	49.4
Death	194	46.1
Narrowing of the female genital tract	155	36.8
Low self-esteem	121	28.7
Emotional distress and depression	108	25.7
**Legality of FGM**		
FGM is being socially discouraged	368	87.4
FGM is against the law in Nigeria	243	57.7

FGM: Female Genital Mutilation, *Multiple responses were allowed

Practice about FGM was assessed using six variables which included having genitally mutilated a female child (Table 2). A correct answer for each of the variables, denoting a behaviour in support of the abolition of the harmful practices regarding FGM, attracted a score of 1 while an incorrect answer, which showed support for perpetuation of the harmful practice was scored 0, giving a total score of 6 marks. A mean score was estimated as a measure of the overall practice about FGM.

**Table 2:** Practices about girl child female genital mutilation among married women

Variable	Frequency n = 421	%
History of being genitally mutilated		
Yes	212	50.4
No	209	49.6
**Respondent’s support for female genital mutilation**		
Yes	60	14.3
No	361	85.7
**Husband’s support for female genital mutilation**		
Yes	74	17.6
No	347	82.4
**Respondent’s support for discrimination against girls who have not undergone female genital mutilation**		
Yes	35	8.3
No	386	91.7
**Ever genitally mutilated a female child**		
Yes	85	20.2
No	336	79.8
**Intention to perform female genital mutilation on a future daughter**		
Yes	31	7.4
No	390	92.6

Factors associated with the practice of FGM were assessed by cross-tabulating independent variables with having ever genitally mutilated a female child or not and having the intention to perform FGM on a future daughter or not.

## Results

Four hundred and twenty-one (99.8%) out of 422 women interviewed responded. The mean age of the respondents is 40.5 ± 14.9 years and majority of them are of the Igbo tribe (97.4%) and practice Christianity (92.2%, Table 3). The majority, (67.2%) were schooled up to the secondary level including 25.2% who had tertiary education. Up to 29.5% of the respondents are unemployed. Similarly, majority (67%) of their husbands attained secondary education including 28% that attained tertiary level and majority (94%) are employed. The majority of the women have female children (83.4%) and approximately half (49.9%) of them belong to the high socioeconomic class with an average monthly family income of N52,881 (USD$119.1). However, 27.6% of them earn less than the N30,000 (< USD$67.6) national minimum wage in Nigeria. Regarding knowledge of FGM, the mean score of the respondents, on a scale of 15, is 8.1 ± 4.3 marks and 51.3% of them achieved average marks (good knowledge).

**Table 3:** Socio-demographic characteristics of respondents and their spouses

Variables	Frequency n = 421	%
Age group in years		
< 30	115	27.3
30–39	111	26.4
40–49	81	19.2
50–59	52	12.4
≥ 60	62	14.7
**Mean age**	40.5 ± 14.9*	
**Tribe**		
Igbo	410	97.4
Yoruba	7	1.7
Hausa	4	1.0
**Religion**		
Christianity	388	92.2
Islam	5	1.2
Traditional	28	6.7
**Level of education**		
No formal education	86	20.4
Primary education	52	12.4
Secondary education	177	42.0
Tertiary education	106	25.2
**Husband education**		
No formal education	83	19.7
Primary education	56	13.3
Secondary education	164	39.0
Tertiary education	118	28.0
**Employment status**		
Unemployed	124	29.5
Self-employed	208	49.4
Salary-employment	89	21.1
**Husband employment status**		
Unemployed	25	5.9
Self-employed	307	72.9
Salary- employment	89	21.1
**Number of female children**		
None	70	16.6
1–4	307	72.9
**≥ 5**	44	10.5
**Family monthly income in the nearest naira ($)**		
< 30,000 (<$72)	116	27.6
30,000–59,000 ($72 - $143)	168	39.9
60,000–89,000 ($145 - $215)	67	15.9
≥ 90,000 ($≥217)	70	16.6
**Mean family monthly income in naira**	52,881 ± 43,532*	
**Socio-economic class**		
Low	211	50.1
High	210	49.9

*Mean ± 1 Standard Deviation; **Tiv,

Table 1 shows that the majority of the respondents are aware of FGM (96.7%), that FGM is a violation of the fundamental human rights of girls and women (59.9%), and that it has no health benefits (56.5%). Further, majority of them are aware that women who are genitally mutilated are at risk of tetanus and sexually transmitted diseases (STDs) such as HIV (53.2%), but only a few of them know that FGM can reduce fertility in women (37.3%) and cause prolonged labour during child-birth (37.8%). The majority of the respondents know that FGM can cause severe bleeding (71.3%) and reduction in sexual feeling (66.3%). Knowledge of the long-term side effects and complications of FGM is poor including narrowing of the female genital tract (36.8%), low self-esteem (28.7%) and emotional distress and depression (25.7%). The majority of the respondents know that FGM is not only being discouraged (87.4%), but it is also against the law in Nigeria (57.7%).

**Fig. 1:**
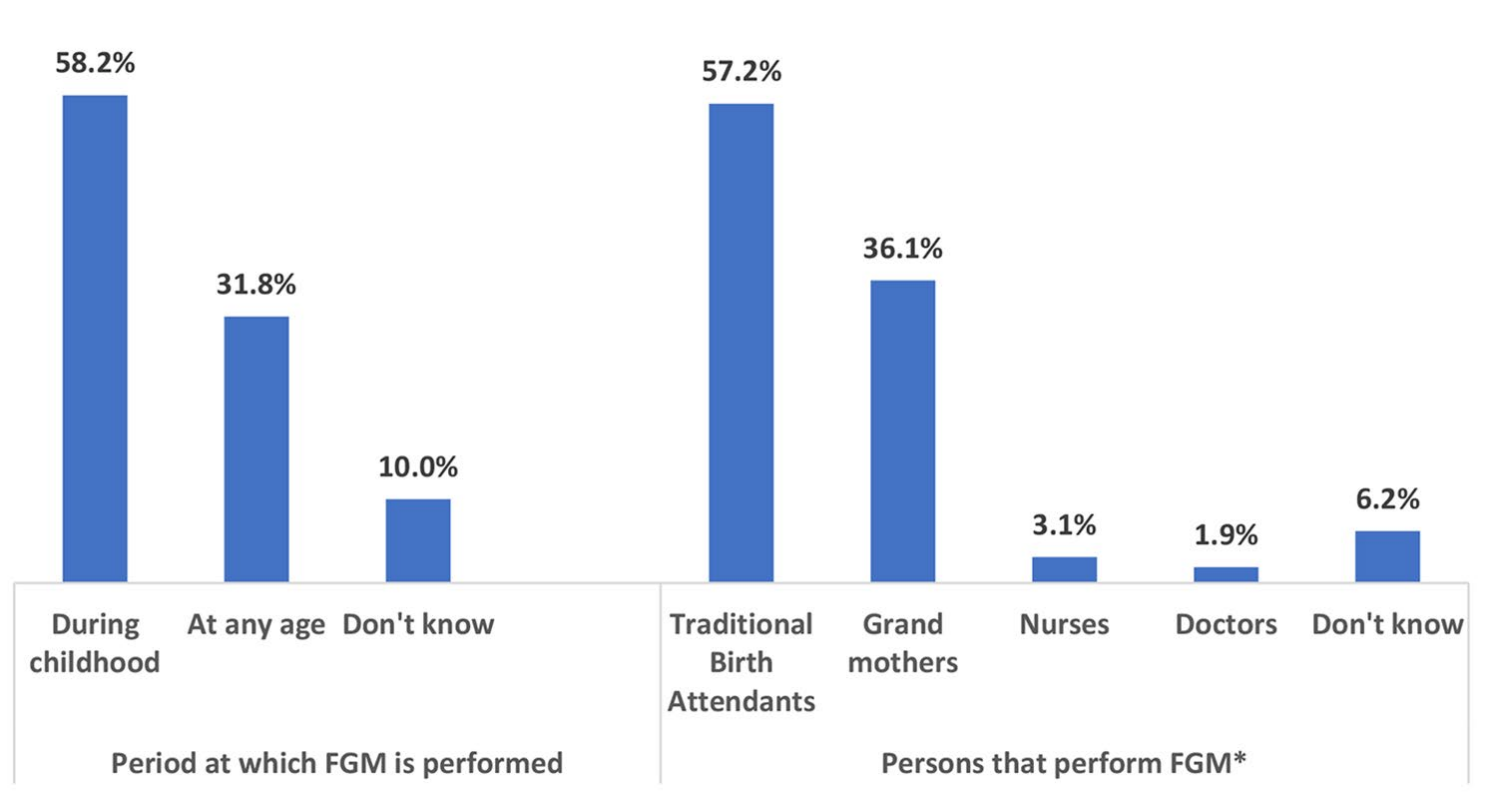
Period at which female genital mutilation is performed and personnel that carry out the procedure *Multiple responses

**Fig. 2:**
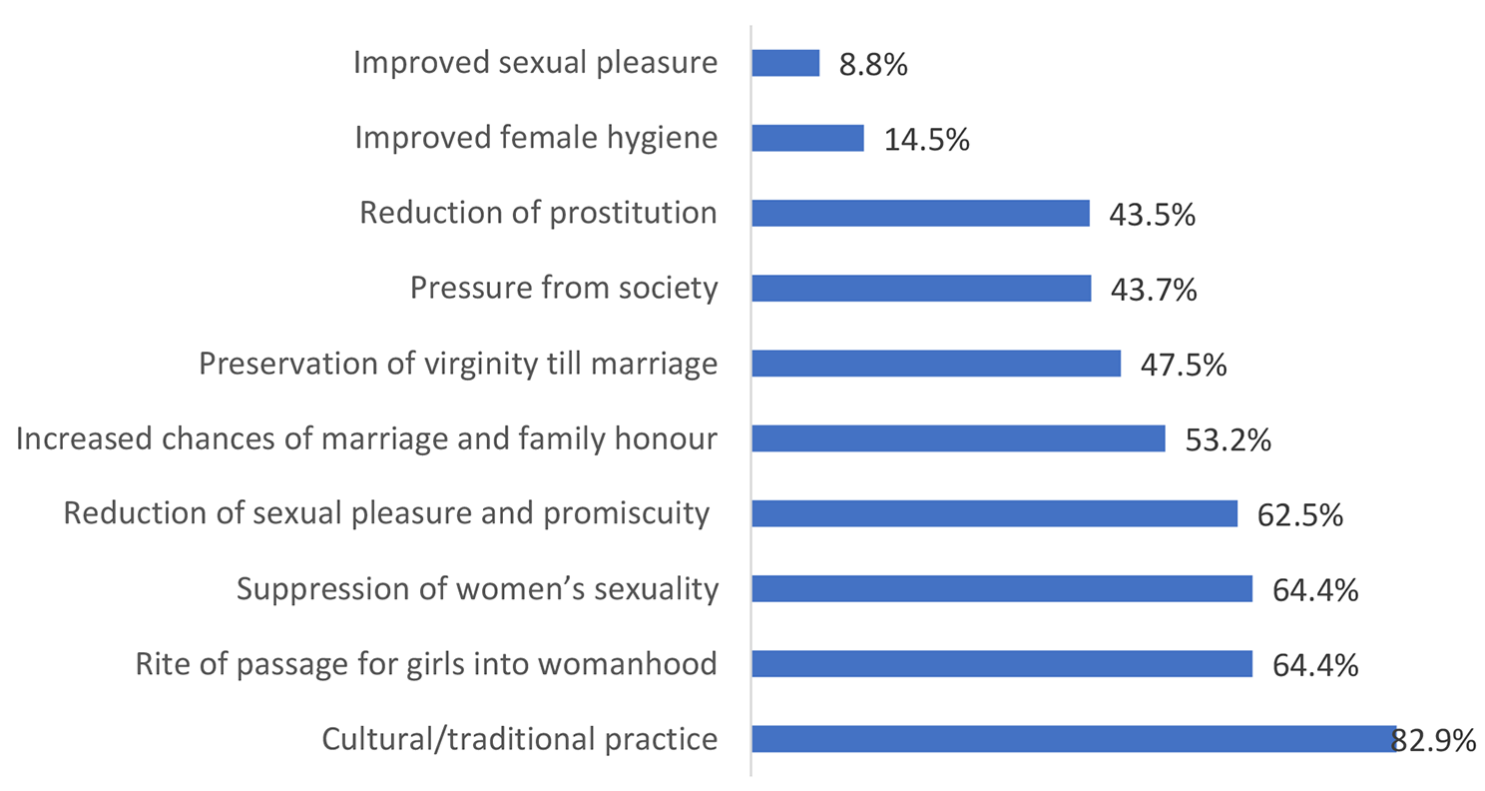
The reasons given by the respondents for Female Genital Mutilation

The majority (85.7%) of the respondents and their husbands (82.4%) do not support FGM (Table 2). Also, majority (91.7%) do not support discrimination against girls who have not been genitally mutilated. Whereas 50.4% of the respondents were genitally mutilated and 20.2% have genitally mutilated their daughters, 7.4% of them have plans to genitally mutilate their future daughters. The mean practice score of the respondents was 4.8 ± 1.2 marks on a scale of 6. Figure 1 shows that FGM is commonly performed by Traditional Birth Attendants (TBAs, 52.7%), mainly during childhood (58.2%). Among reasons adduced for performing FGM, tradition (82.9%), rite of passage of girls into womanhood (64.4%), suppression of women’s sexuality (64.4%), reduction of sexual pleasure and promiscuity (62.5%) and increased chances of marriage and family honour (53.2%) were topmost (Fig. 2).

The factors associated with having genitally mutilated a female child are presented in Table 4. Age, respondent and husband education, employment status, number of female children, socio-economic class, being genitally mutilated, and knowledge of FGM are significantly associated with having genitally mutilated a female child. Women who attained secondary education have four times less genitally mutilated their female children compared to those who did not have formal education (Adjusted Odds Ratio [AOR] = 0.248, 95% Confidence Interval [CI] = 0.094–0.652). On the other hand, women who are employed have over four and half times, more genitally mutilated their female children compared to those who are unemployed (AOR = 4.723, 95% CI = 1.881–11.859), and women who were genitally mutilated have approximately 29-times more genitally mutilated their female children compared to those who are not genitally mutilated (AOR = 28.732, 95% CI = 6.171-133.768, Table 4).

**Table 4:** Factors associated with genital mutilation of female child among married women in Abakaliki

Independent Variable	Ever genitally mutilated a female child		Bivariate Analysis	Logistic Regression	
	No (N = 336) n (%)	Yes (N = 85) n (%)	χ^2^ (p-value)	AOR	95% CI for AOR
Age in years					
< 40	215 (64.0)	11 (12.9)	71.095 (< 0.001*)	1	
≥ 40	121 (36.0)	74 (87.1)		1.358	0.529–3.483
**Level of education**					
<Secondary	68 (20.2)	70 (82.4)	118.786 (< 0.001*)	1	
≥Secondary	268 (79.8)	15 (17.6)		0.248	0.094–0.652*
**Husband education**					
<Secondary	70 (20.8)	69 (81.2)	111.695 (< 0.001*)	1	
≥Secondary	266 (79.2)	16 (18.8)		0.432	0.162–1.152
**Employment status**					
Unemployed	112 (33.3)	12 (14.1)	12.055 (0.001*)	1	
Employed	224 (66.7)	73 (85.9)		4.723	1.881–11.859*
**Husband employment status**					
Unemployed	23 (6.8)	2 (2.4)	2.451 (0.117)	NA	NA
Employed	313 (93.2)	83 (97.6)			
**Number of female children**					
None	68 (20.2)	2 (2.4)	45.460 (0.001*)	1	
1–4	248 (73.8)	59 (69.4)		1.092	0.167–7.142
≥ 5	20 (6.0)	24 (28.2)		2.605	0.329–20.615
**Socio-economic class**					
Low	137 (40.8)	74 (87.1)	58.133 (< 0.001*)	1	
High	199 (59.2)	11 (12.9)		0.428	0.168–1.093
**History of being genitally mutilated**					
No	207 (61.6)	2 (2.4)	95.279 (< 0.001*)	1	
Yes	129 (38.4)	83 (97.6)		28.732	6.171-133.768*
**Knowledge of Female Genital Mutilation**					
Poor^**^**^	132 (39.3)	73 (85.9)	58.958 (< 0.001*)	1	
Good^**^^**^	204 (60.7)	12 (14.1)		0.532	0.228–1.244

**AOR**: Adjusted Odds Ratio; **CI**: Confidence Interval; *Statistical significance; ^Below mean score; ^^Mean score and above

Table 5 shows the factors associated with the intention to genitally mutilate a future daughter. It is shown that age, respondent and husband education, employment status, number of female children, socio-economic class, being genitally mutilated, knowledge of FGM, and having genitally mutilated a female child are significantly associated with the intention to genitally mutilate a future daughter. Women who have genitally mutilated a female child have approximately 142 times greater propensity to mutilate their next daughters compared to those who have not genitally mutilated a female child (AOR = 141.786; 95% CI = 9.584-209.592, Table 5).

**Table 5:** Factors associated with the intention to perform female genital mutilation on a girl child among married women in Abakaliki

Independent Variable	Intention to genitally mutilate a future daughter		Bivariate Analysis	Logistic Regression	
	No (N = 390) n (%)	Yes (N = 31) n (%)	χ^2^ (p-value)	AOR	95% CI for AOR
Age in years					
< 40	220 (56.4)	6 (19.4)	15.859 (< 0.001*)	1	
≥ 40	170 (43.6)	25 (80.6)		0.862	0.179–4.158
**Level of education**					
<Secondary	112 (28.7)	26 (83.9)	39.644 (< 0.001*)	1	
≥Secondary	278 (71.3)	5 (16.1)		0.537	0.108–2.677
**Husband education**					
<Secondary	114 (29.2)	25 (80.6)	34.325 (< 0.001*)	1	
≥Secondary	276 (70.8)	6 (19.4)		0.746	0.149–3.745
**Employment status**					
Unemployed	119 (30.5)	5 (16.1)	2.859 (0.091)	1	
Employed	271 (69.5)	26 (83.9)		1.346	0.389–4.660
**Husband employment status**					
Unemployed	25 (6.4)	0 (0.0)	2.113 (0.146)	NA	NA
Employed	365 (93.6)	31 (100)			
**Number of female children**					
None	68 (17.4)	2 (6.5)	29.153 (0.001*)	1	
1–4	290 (74.4)	17 (54.8)		0.328	0.043–2.489
≥ 5	32 (8.2)	12 (38.7)		0.756	0.081–7.091
**Socio-economic class**					
Low	184 (47.2)	27 (87.1)	18.303 (< 0.001*)	1	
High	206 (52.8)	4 (12.9)		1.046	0.222–4.938
**History of being genitally mutilated**					
No	205 (52.6)	4 (12.9)	18.070 (< 0.001*)	1	
Yes	185 (47.4)	27 (87.1)		0.128	0.008–2.078
**Knowledge of Female Genital Mutilation**					
Poor^**^**^	178 (45.6)	27 (87.1)	19.755 (< 0.001*)	1	
Good^**^^**^	212 (54.4)	4 (12.9)		0.600	0.141–2.549
**Ever genitally mutilated a female child**					
No	333 (85.4)	3 (9.7)	102.147 (< 0.001*)	1	
Yes	57 (14.6)	28 (90.3)		141.786	9.584-209.592*

**AOR**: Adjusted Odds Ratio; **CI**: Confidence Interval; *Statistical significance; ^Below mean score; ^^Mean score and above

## Discussion

This study sought to determine the factors associated with the practice and the intention to perform FGM and found that half of the respondents were genitally mutilated, one fifth of them have genitally mutilated their daughters and some of them still have the intention to do same to their next female children. This study further showed that whereas education promotes abandonment of FGM, women who underwent FGM have a greater propensity to perpetuate the practice. A previous survey in Nigeria had shown a similar pattern whereby women who are genitally mutilated are more likely to believe that FGM should be continued [7]. Also in Egypt, majority of a survey respondents who were genitally mutilated did not see any harm from being genitally mutilated [25]. This observation suggests a victim-precipitated action and highlights a need for targeted interventions to dispel any perceived sexual or health benefits of FGM. Further, the reasons for this seeming greater support for FGM among this group of women needs to be carefully investigated. The reasons adduced by our respondents for performing FGM align with previous reports [1, 5, 9–11] which revolves around beliefs in socio-cultural, sexual and health benefits of FGM. These reasons, however, do not justify the subjection of girls and women to the painful consequences of FGM. Strategies need to be carefully designed to respect the cultural values of the practice within the communities where FGM is performed while upholding the human rights of girls and women [9].

The prevalence of FGM in our study is slightly lower than the reported for Ebonyi State in the NDHS 2018 [7] and much lower than previous report in Abakaliki by Lawani et al. [8]. The discrepancy between our findings and previous ones may be due to improvement in the abandonment of the practice or the fact that Lawani et al. surveyed women in specialist obstetric facilities in Abakaliki metropolis; those women may have visited the facilities from far and wide places to access special gynaecologic and obstetric services due to complications, including those that may have arisen from FGM. Our finding is much higher than the 27.1% reported in Bayelsa State of Nigeria [26], and 14.7% among Egyptian medical students [25], but much lower than 91.7% in Ethiopia [15], and 70% among African women who had migrated to Canada [27]. These discrepancies may be explained by changing trends in the practice of FGM or differences in socio-cultural, traditional, or religious backgrounds, based on which the practice of FGM has been defended [9–11].

The poor knowledge of some long-term complications of FGM among the respondents is a cause of concern. Similarly, it was reported in NDHS 2018 that only 61% of women in Nigeria have heard of FGM [7]. This finding suggests the need for public education to increase knowledge of FGM and its consequences and positively influence behaviours towards its eradication. Notwithstanding that FGM remains prevalent in certain countries where laws against it exist [3], it is interesting that majority of our respondents know that it is not just a violation of fundamental human right, it is now a punishable offence in Nigeria, prescribed in existing federal and state laws banning it [5, 18, 19]. These laws are expected to serve as a deterrent to the proponents of FGM. The fact that Ebonyi State Violence Against Persons (Prohibition) Law established the FGM Monitoring Committee (FGMMC) at the State, LGA, and Community levels [19] is praiseworthy; however, a challenge that may not be disputed is that public awareness of the legislations is not optimal. This suggests the need for expanded enlightenment campaigns about the existing laws and the penalties specified against FGM. In line with our findings, such campaigns should be designed to target women who were genitally mutilated and those who have genitally mutilated their daughters or supported someone to engage in the harmful practice, since they have a greater propensity to perpetuate the practice. Such targeted interventions will require experts in gender-sensitive matters like FGM who will not only be able to identify the victims of FGM but also be empathic enough towards them and able to positively influence their disposition on the matter, helping them to dispel any harboured erroneous beliefs about FGM.

The fact that FGM is commonly performed by TBAs has previously been reported in Nigeria [2, 28]. Consequently, there is a need for regulation of informal health service providers and traditional medicine practitioners who are at the forefront of the harmful practice, including placing and enforcing sanctions against any offenders. Though our study shows that only 5% of FGM are performed by doctors and nurses, a procedure known as medicalization [1], reports of medicalization abound in Nigeria [26]. Experts estimate that only 18% of women who have been subjected to FGM had the procedure performed by trained healthcare personnel [29]. The prevalence of medicalization in our study is slightly lower than the 7.6% reported in a study done in Gambia [30]. Medicalization had been advanced to reduce the complications of FGM because trained medical personnel are skilled to carry out the procedure in a sterile environment and condition to avoid infections and manage any immediate consequences such as bleeding. However, there is the risk of immediate and long-term consequences even when the procedure is performed in a sterile environment by a healthcare provider [31–33]. Further, FGM has no medical justification, violates the code of medical ethics, and the rights to health, life, physical integrity, and non-discrimination, and the rights to be free from cruel, inhuman, or degrading treatment [31, 32]. On a larger note, medicalization may confer a sense of legitimacy to FGM or give the impression that it is without health consequences, which can undermine global efforts towards its eradication [32]. These facts make it imperative for the Medical and Dental Council and the Nursing and midwifery Council of Nigeria to enforce sanctions against medical and nursing and midwifery practitioners who perform FGM, in a bit to discourage the harmful practice.

Similar to our finding, previous studies have shown that women who attained secondary education are less likely to genitally mutilate their daughters [34] or support FGM [6, 27]. Education is an important mechanism to increase awareness of the dangers of FGM, foster questioning and, discussion and provide opportunities for individuals to take on social roles that are not dependent on the practice of FGM for acceptance [34]. Based on this fact, the design of interventions towards the abandonment of FGM must take the role of women’s education into cognizance, making girl child education a priority in all states of Nigeria and countries in Africa where FGM is rampant.

The NDHS 2018 showed that the prevalence of FGM declined by 5% compared to 2013 [7]; this rate is small. There is a need for sustained enforcement of the legislations about FGM to ensure that Nigeria meets the SDG 5 target 5.3 by 2030 [17]. The efforts to stop FGM must focus on human rights, gender equality, sexual education, and attention to the needs of women and girls who suffer from its consequences [35].

## Conclusion

The practice of FGM remains high and is worse among women who underwent FGM; they also have a greater propensity to perpetuate the harmful practice into their next female children. This finding suggests a victim-precipitated action due to harboured erroneous beliefs in the socio-cultural, sexual, and health benefits of FGM. The reasons behind the seeming greater support for FGM among victims of the harmful practice needs to be carefully investigated. In the meantime, interventions must target these victims to change their perception and help them to dispel such erroneous beliefs. Although the reasons for practice of FGM have persisted, they do not justify the subjection of girls and women to the pains of FGM and the negative long term consequences. Education is an important factor for the abandonment of FGM; any interventions must take the role of women’s education into cognizance, making girl child education a priority in societies where it is practiced. Further, the need for improved legislation with enforcement of sanctions against FGM to serve as a deterrent to offenders cannot be overemphasized.

### Limitations of the study

Female genital mutilation is a gender-sensitive matter associated with emotional trauma, stigmatization and discrimination in the victims. Further, there has been increasing legislations against FGM with prescriptions of punitive measures against perpetuators and supporters of the harmful practice. As a result of these, victims of FGM and individuals who perpetuate the practice are most likely to be secretive and biased against reporting their experiences and practices about FGM. Consequently, inadequate responses from our participants could have affected the conclusions drawn from this study. However, the researchers were mindful of these potential limitations and remained civil and empathic while interviewing the respondents during the data collection. The researchers also assured the respondents of the confidentiality of the information they provided.

## Data Availability

All relevant data are within the manuscript. The data supporting these findings can also be made available upon request to the corresponding author.

## List of Abbreviations

AOR: Adjusted Odds Ratio
CI: Confidence Interval
FGM: Female Genital Mutilation
FGMMC: Female Genital Mutilation Monitoring Committee
LGA: Local Government Area
NDHS: Nigeria Demographic and Health Survey
SDG: Sustainable Development Goal
STD: Sexually Transmitted Diseases
TBA: Traditional Birth Attendants
USD: United States Dollars
WHO: World Health Organization
